# Wild sage (*Salvia officinalis*) as a potential anti-malarial drug

**DOI:** 10.1186/1475-2875-11-S1-P3

**Published:** 2012-10-15

**Authors:** Mutaz Akkawi, Abd-Alkarem Sharif, Khaled Salem, Azzam Saleh, Qasem AbuRemeleh

**Affiliations:** 1 Department of Life Sciences, College of Science and Technology, Al-Quds University, 91999, Palestine

## Background

During the intra-erythrocytic stage, *Plasmodium* parasites degrade hemoglobin resulting in ferriprotoporphyrin (IX) accumulation; toxic to the parasite [1].

β- Hematin, a synthetic polymer made from ferriprotoporphyrin- IX is structurally, chemically and spectroscopically identical to purified hemozoin is used in *in-vitro* studies [1].

Resistance to Chloroquine, highlights the need for new drugs. Earlier attempts showed the effect of pyrimidine derivatives in *in-vitro* inhibition of β-hematin [2], and cisplatin complexes [3]. We concentrate on finding new molecules from natural products; (*Salvia officinalis*)*.*

## Materials and methods

Plant materials, collected from areas around Jerusalem, were dried at room temperature; leaves and stems separately grinded. Extraction was by soaking 5gm of dried plant parts in 40 ml of 35% ethanol or ultrapure -water; left standing for about 24-hours. Extracts were then filtered using MN615- Φ 90 nm filter paper, rotary evaporated at 50°C then lyophilized to constant weight. Stock solutions were prepared in water.

## Semi-quantitative method

The procedure was according to [4], ultra-pure water for negative control, chloroquine or *Amodiaquine* for positive control. The final precipitate of β-hematin dissolved in 200µl of 0.1 M NaOH to give alkaline hematin for spectroscopic quantification at 405 nm.

## Results

The efficiency of sage leaf extracts compared to controls is shown below. Each absorption value is the average of five experiments. The mechanism of inhibition is probably through formation of a complex between active compounds in these extracts and ferriheme; this complex prevents the formation of β-hematin.

**Table 1:** 

	Absorbance				average
Negative Control (H2O)	2.26	2.14	2.05	2.10	2.137
Negative control 35% ethanol	2.22	2.01	2.21	2.23	2.167
Chloroquine 0.1 mg/ml	0.045	0.047	0.055	0.179	0.071
Amodiaquine 0.1 mg/ml	0.058	0.055	0.066	0.048	0.056
Chloroquine in 35% ethanol 0.1 mg/ml	0.055	0.062	0.055	0.059	0.057
Stock-leaf extract in 35% ethanol 1 mg/ml	0.060	0.057	0.047	0.069	0.058
Stock-leaf extract in 35% ethanol 0.5 mg/ml	0.044	0.074	0.059	0.060	0.059
Stock-leaf extract in water 1 mg/ml	0.099	0.080	0.085	0.110	0.093
Stock-leaf extract in water 0.5 mg/ml	0.378	0.162	0.270	0.255	0.266

## Conclusion

The extract is a natural product and has been used in folk medicine with no reported toxicity.

Although the results for the extracts are lower than for the positive controls we must take into account the fact that the extracts are crude, we are already working on isolating the ingredients; results will be published in the near future.
